# TRYMS: the ethical and practical considerations of a double-blind placebo controlled randomised clinical trial

**DOI:** 10.1186/1745-6215-16-S2-P200

**Published:** 2015-11-16

**Authors:** Jayne Swain, Isabelle Smith, Alexandra Smith, Jennifer Walsh, Richard Ross, Helen Marshall

**Affiliations:** 1 Clinical Trials Research Unit, Leeds Institute of Clinical Trials Research, Leeds, UK; 2 University of Sheffield, Sheffield, UK

## 

A double blind randomised trial is the gold standard approach to minimise the introduction of bias from trial participants, healthcare professionals and clinical trial staff. Similarly, the use of a placebo is ideal when participant self-reported Quality of Life is one of the primary objectives. However, the combination of these trial design components can present challenging ethical and practical issues for good trial conduct.

TRYMS is a randomised, double-blinded, placebo controlled phase IV clinical trial in young male cancer survivors with low testosterone levels. The trial is designed to investigate whether testosterone treatment will result in a reduction of truncal fat mass and an increase in participant self-reported physical functioning scores and recruited 136 participants from 10 centres across the UK.

Practical issues relate to the potential for centre and trials unit staff to be unblinded by access to clinical data: for example, retests of testosterone measurements required for dose titration were required in the active arm but were done in the placebo arm to maintain the blind. Other issues relate to managing Investigational Medicinal Product supply requirements: a minimisation programme is used to ensure treatment groups are well-balanced by randomising centre however, a random element and participant replenishment halfway during treatment can result in a temporary skew to a centre’s IMP requirements. The ethical issues relate to non-emergency requests for unblinding due to participant perception of treatment allocation.

Our experiences in TRYMS, along with our solutions, will be presented with considerations for the management of similar trials.

